# Investigation of the acoustic agglomeration on ultrafine particles chamber built into the exhaust system of an internal combustion engine from renewable fuel mixture and diesel

**DOI:** 10.1016/j.heliyon.2023.e16737

**Published:** 2023-05-29

**Authors:** Kristina Kilikevičienė, Aleksandras Chlebnikovas, Jonas Matijošius, Artūras Kilikevičius

**Affiliations:** Institute of Mechanical Science, Vilnius Gediminas Technical University, Plytinės str. 25, LT-10105 Vilnius, Lithuania

**Keywords:** Acoustic agglomeration, Engine exhaust, Fractional numerical concentration of particulates, ROMEP fuel Mixture

## Abstract

Reducing the pollution of internal combustion engines is a very important problem that can be solved in various ways. However, the acoustic agglomeration method is not used in diesel engines. The study used a 1.9 TDI diesel internal combustion engine supplied with a mixture of diesel (D100) and a 90% of rapeseed methyl ester - 10% propanol fuel mixture (ROMEP). The study also changed the position of the exhaust gas recirculation (EGR) valve by adjusting the 20% EGR throughput limits and maintaining a constant engine load of 90 Nm. It should be noted that the use of biofuels produces less particulate matter, which reinforces the relevance of this study. Measurements were performed using Measurement System: The Testo 380 fine particle analyzer system was used to determine the mass concentration, and a six-channel Fluke 985 particle counter with an isokinetic sampling probe was used to determine the fractional numerical concentration of the particulates. Six particle size distribution regimes in the size range of 0.3 to 10 μm were observed, controlling the transmittance of the EGR system by 20%. The direction of the sound pressure throughout the flow and the excitation frequency 21400 Hz and 33800 Hz were also investigated and compared with the results without agglomeration. The article examines the possibility of using the developed acoustic chamber in the exhaust systems of various objects that uses diesel or various alternative fuel mixtures as fuel. The acoustic field reduces the number of particles by up to 92.5% for 10 μm and up to 44.5% for 0.3 μm at an excitation frequency of 21400 Hz.

## Nomenclature

D100: type of fuel consisting of 100% diesel
ROMEP: type of fuel consisting of 90% of rapeseed methyl ester and 10% of propanol
EGR: exhaust gas recirculation

**Symbols**
PM: particulate matter
PM2.5: fine particulate matter is defined as particles that are 2.5 μm in diameter or less in diameter
UFP: ultra-fine particles
NOx: nitrogen oxides
TDI: turbo diesel inject
CO: carbon monoxide;
O2: molecular oxygen
NO_2_: nitrogen dioxide;
NO: nitrogen monoxide;
ECU: electronic engine control unit
EGR: exhaust gas recirculation system
SO_2_: sulfur dioxide;
ppm: particle per million parts

## 1 Introduction

Various treatment plants and systems are being developed to remove harmful particles from the environment. One possible solution is electrostatic filtration systems, where the particles in the clean air stream are exposed to a direct electric current, receive an electric charge, and are deposited on a surface with the opposite charge. However, electrostatic precipitation systems are more efficient for collecting particles larger than 1.0 μm in diameter and very low removal efficiency for smaller particles [[1], [2], [3], [4], [5]]. Therefore, ways to combine the solids into larger agglomerates and take advantage of the electrostatic precipitator are sought. One such method is acoustic agglomeration, that is, when particles in the purified air stream are exposed to a high-intensity acoustic field and the fine particles agglomerate and grow into larger ones [6,7].

One of the most advanced acoustic agglomeration research methods is increasingly used to enlarge aerosol particles, which is increasingly used in the work of researchers [[8], [9], [10]]. When defining the mechanism of acoustic agglomeration, the following stages can be distinguished: sound waves affected by aerosol particles move relatively, as a result of which the probability of their collision increases; this causes the particles to grow larger as smaller particles collide with each other, and as the size of the overall particle grows, this process becomes continuous as the newly formed particles collide with each other again and thus grow larger. Hydrodynamic interaction and orthokinetic collision mechanisms are distinguished in the literature when examining the agglomeration mechanism.

Acoustic agglomeration, or known in the literature as ultrasonic agglomeration, is described as a process that occurs when suspended particles stick together in a high-intensity aerosol acoustic field. In addition to the hydrodynamic interaction and orthokinetic collision mentioned above, which are predominant, there are other factors that strengthen these processes, such as the effects of turbulence, radiation force, and acoustic flow. It is noted that the following conditions are necessary for the orthokinetic interaction to work: the distance between the suspended particles must be the same as the displacement amplitude of the sound field, and the ensured movement of these particles must be parallel to the direction of the sound source. When these conditions are met, particle vibrations of different phases and amplitudes are obtained due to inertial forces and differential flow parameters. Such an activated movement of particles ensures a higher probability of collision between them, and at the same time, the execution of the agglomeration process. It is clear that hydrodynamic forces also act in the agglomeration medium, which allows the particles to increase in size larger than their acoustic displacements. Then the orthokinetic mechanism is eliminated due to similarly behaving particles in the acoustic field, i.e., there is no direct collisional effect because the interaction occurs in monodisperse media. This promotes an acoustic wake effect.

Acoustic agglomeration can be used as a pre-treatment technology before particles enter conventional dust removal facilities. High-intensity sound waves promote the vibration of particles of different sizes in the medium at different amplitudes and velocities, and due to the relative movement between particles, they collide and agglomerate more easily [[11], [12], [13], [14], [15]]. These larger particles are constantly in contact with others and after some time the particle size distribution shifts towards larger sizes. Therefore, the further dust removal process becomes more efficient.

There are some essential gaps in the implementation of acoustic agglomeration. The particle agglomeration was applied to impact the dusty gas flow from the medium and small boilers, but not for a flue gas flow from the internal combustion engine using fossil and renewable fuels and their mixtures. Also, there is research on agglomeration due to acoustic impact using modified source of pollution from the combustion process of rapeseed methyl ester distinguished from the others by the emission of particularly ultra fine particulate matter.

The emissions of micron and submicron particulate matter from fossil fuel combustion are recognized as a serious environmental problem. These particles, especially PM 2.5, stay in the air for long periods of time and have been shown to pose a health risk by often becoming carriers of various viruses and bacteria that easily enter the human body through respiration. Although many particulate removal technologies have been developed, such as electrostatic precipitators, high-efficiency filters, etc., those technologies are still inefficient in removing PM 2.5. Therefore, it is necessary to develop an aerosol agglomeration method that could cause microparticle coagulation and help improve the removal efficiency of PM 2.5 removal technologies [[16], [17], [18]].

Car flows, heavy industry, can be a major source of urban air pollution and can therefore affect local and regional air quality [[19], [20], [21]].

Several studies [[22], [23], [24]] emphasize that the most important thing is to evaluate extremely small particles (i.e., ultra-small particles (UFPs) (Dp < 100 nm)) for their effects on human health. These particles can penetrate deeper into the respiratory system than larger particles. The larger particles settle in the upper respiratory mucus layer, that is, in the nose and throat. UFP enters the lungs, resulting in a higher deposition efficiency in the lungs than in larger particles. These particles travel through the blood to other organs and tissues. Studies in humans and animals highlight the effects of UFP on the respiratory and cardiovascular systems, with changes in lung function, an increased allergic response, and changes in heart rhythm observed. In addition, a link between particles and intestinal diseases is believed to exist.

Pollution from mobile pollution sources is one of the most pressing environmental problems today. Considering the fact that transport is one of the main generators of this pollution, it can be said that its heterogeneous pollution consists of both solid particles and sulfur, hydrocarbons, and other compounds. Consequently, differences in each pollution component will manifest both in the size of the particle and in its toxicity and concentration. It is emphasized that the greatest effect on human health is determined by solid particles released during diesel combustion [25].

The principle of action of the agglomerator is based on the effect of sound, that is, a wave of increased pressure. On the basis of the theory of chemical equilibrium, it is known that when pressure is increased, chemical reactions shift to the side where there is a smaller volume of substances without taking solids. Thus, if a reversible reaction is formed of primary substances, mostly consisting of solids, resulting in gaseous substances, the effect of the agglomerator will cause the reaction path to be reversed. At constant temperature and volume, the pressure will depend only on changes in the number of moles. If the amount increases, the pressures will increase and vice versa [26]. However, if we assume that the equilibrium in the system is constant, then the change in pressure is directly proportional to the ratio of the changing temperature and volume of the initial substances and reaction products [27]. Thus, we can assume that if the pressure is increased by the sound wave created by the agglomerator, and the ratio of temperature to volume remains the same, the molar concentration should change, and hence the reaction will shift in the opposite direction. In the case of combustion, this creates conditions for decreasing the concentration of oxides and vice versa increasing the oxygen content [28].

Emissions of particulate matter, NOx, and volatile organic compounds from cars contribute to the formation of ozone [[29], [30], [31], [32]]. The particle size, that is, diameter (Dp) and their chemical composition are important properties in assessing their effects on human health. Many of these particles are volatile and, depending on temperature and other conditions, may remain in the gas phase, condense in existing particulate matter, or nucleate and form new particles [[33], [34], [35]]. Typical diesel particles are agglomerates composed mainly of primary spherical particles with a diameter of 15 to 40 nm [36,37].

This research aimed to determine the effect of acoustic agglomeration (at ultrasonic wavelengths, up to 35 kHz in this study) on the composition of pollutants in the gas stream, including the effect of wavelength on the agglomeration of particles with different dispersion, by means of an acoustic field generated by different characteristics, for the combustion of fossil fuels, renewable fuels, and mixtures of them.

Future studies are foreseen to investigate the pollution components and acoustic agglomeration effects of other fully renewable fuels; Experimental studies on the sequential application of acoustic agglomeration and particle settling technology. Determine the particle removal efficiency depending on the parameters of the acoustic field, the type of fuel, and the operating mode of the internal combustion engine.

## 2 Materials and methods

**Fig. 1:**
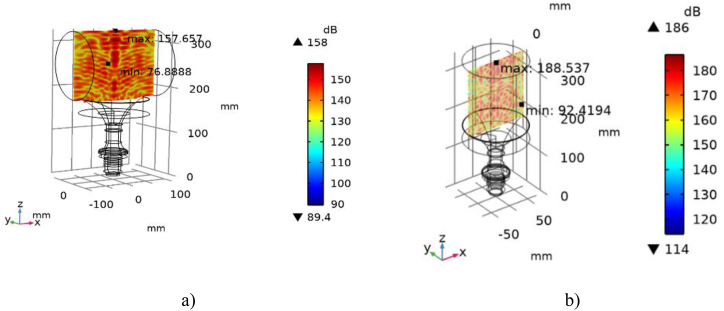
Far-field sound pressure level, dB: a) transverse direction of agglomeration; b) longitudinal direction of agglomeration.

On the basis of Vilnius Tech (Vilnius Gediminas Technical University) laboratory base, an experimental research stand was developed to study the agglomeration of particulate matter in diesel engine exhaust. During the tests, diesel engine emissions were exposed to a sound pressure level of 140 ± 1.0 dB in the acoustic chamber. An acoustic chamber was designed to study the acoustic agglomeration of the particles. The acoustic chamber consisted of a piezoelectric excitation source and angular and longitudinal chambers. These elements were designed and optimized using the Comsol multiphysic software package. The design goal is to achieve a sound pressure level of at least 140 dB in the acoustic chamber, the results are shown in Fig. 1. On the basis of these calculations, an acoustic chamber was fabricated.

The following fuels were used in experimental studies of diesel engine exhaust emissions under acoustic effects: pure fossil diesel (D100) and a mixture of Rapeseed Oil Methyl Esters 90% and 10% Propanol (ROMEP). The physical and chemical properties of the fuel were tested in the laboratory, and their properties are given in Table 1.

**Table 1:** The main physical and chemical properties of the fuels.

Fuel/Parameter	Density (kg/m^3^)	Viscosity (mPa·s) at 40 °C	Mass Fraction (%):			Lower Heat Value, MJ/kg	Cetane number
			Carbon	Hydrogen	Oxygen		
Pure fossil diesel (D100)	825	2.352	86.3	13.7	0	43.97	45
Rapeseed Oil Methyl Esters 90% and 10% Propanol (ROMEP)	869	3.331	75.75	11.8	12.45	36.77	47

Efforts have been made to select a mixture of alternative fuels so that its properties are close to those of fossil diesel fuel. The individual components of the mixture have different properties, so the addition of propanol to the rapeseed methyl ester was defined at a concentration of 10%.

For the tests, a 1.9 TDI turbocharged engine with direct fuel injection (diesel) was used. The engine fuel system is equipped with a distributor type fuel pump controlled by an electronic engine control unit (ECU) and single-injected fuel injectors. The engine is equipped with an exhaust gas recirculation (EGR) system and the intake air is cooled by an air cooler. The main parameters of the 1.9 TDI engine with compression ignition are shown in the works of other authors [34,38,39].

**Fig. 2:**
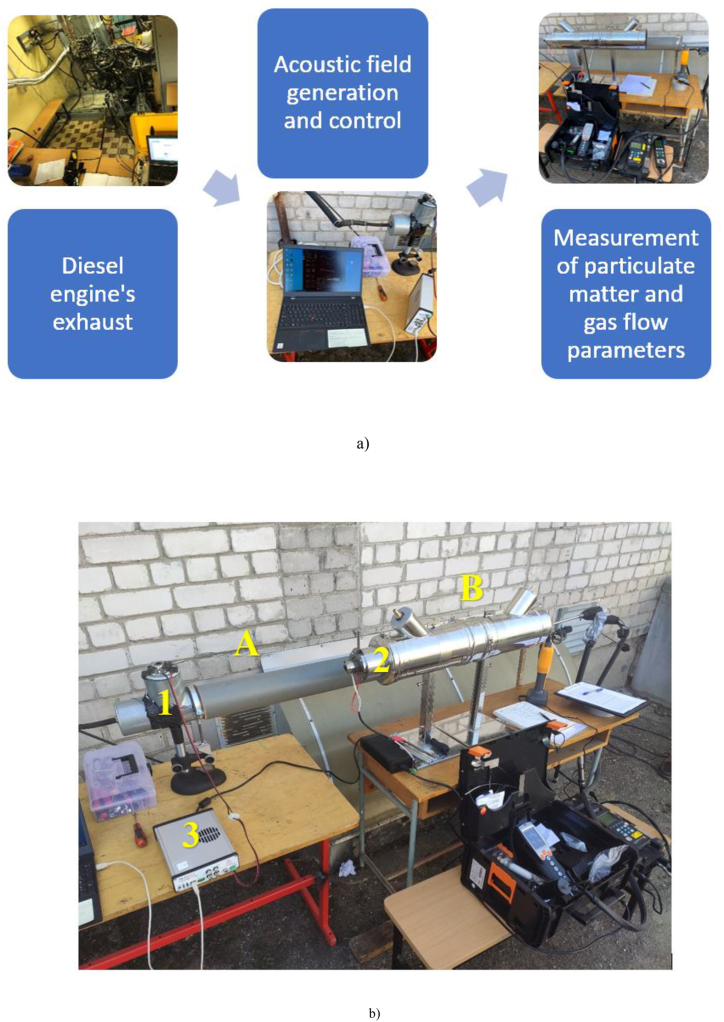
Scheme of experimental tests (a) and experimental stands (b) with acoustic agglomeration control system, equipment to measure gas flow parameters and gaseous pollutants and particulates: 1 - single transverse agglomeration stand, 2 - single longitudinal agglomeration stand.

An acoustic camera developed by VilniusTech researchers (positions 1 and 2 in Fig. 1a) was used to generate the acoustic effect, in which the piezoceramic sound pressure generation device was controlled using a PC and PDUS210 ultrasonic driver (position 3 in Fig. 2). The level of sound pressure was maintained at 140 ± 1.0 dB throughout the tests using an acoustic chamber. For diesel fuel tests, the direction of the sound pressure generated in the acoustic chamber was transverse to the flow (Fig. 2, Part A), and for the tests with the ROMEP fuel mixture, the direction of the sound pressure was generated along the flow (Fig. 2, Part B).

Sample points for exhaust gas flow parameters were installed on the experimental test benches.

The Multifunction Gas Flow Analyzer Testo-350 was used. Limits for the determination of gaseous pollutants: O_2_-0 to 25%; CO-0 to 10,000 ppm; NO-0 to 3000 ppm; NO_2_-0 to 500 ppm. Gas flow temperature was recorded on a thermocouple gas sampling probe. Test: Temperature measurement range: −20 to +350 °C; error - ± 0.2 °C. The gas flow rate in the conduit was determined using a Testo thermocouple connected to a Testo 400 multifunction meter: measuring range 0.01–15 m/s; error - ± 0.01 m/s.

The Testo 380 fine particle analyzer system was used to determine the mass concentration. Particulate measurement: measuring range 0–0.3 g/m^3^, accuracy meets VDI 4206-2, resolution 0.0001 g/m^3^. Gas flow temperature - from 0 to 500 °C, accuracy meets VDI 4206-1 requirements, resolution - 0.1 °C.

A six-channel particle counter Fluke 985 with an isokinetic sampling probe was used to determine the fractional numerical concentration of the particulates. The device particle size range is (0.3, 0.5, 1.0, 2.0, 5.0 and 10.0) μm. The sample flow rate is 0.1 cfm (corresponding to 2.83 l/min). A light source from 775 to 795 nm, 90 mW Class 38 laser is used. Efficiency calculation - 50% for particles larger than 0.3 μm, 100% for particles larger than 0.45 (m (according to ISO 21501). The limit of quantification is 10% at 4 million. ft^3^ (according to ISO 21501). The studies were carried out using cumulative mode. Before each measurement, the instrument shall be calibrated with a zero filter check to an accuracy of not more than 1 particle over a sampling period of 5 (according to JIS 89921).

The tests were carried out in 6 replicates and the results are shown as the mean value.

In this study, the internal combustion engine is used as a particulate pollution generator, and the focus of the study is on agglomeration methods to reduce particulate emissions. From a scientific point of view, there are interesting results for some alternative fuels, and their pollution is compared with fossil diesel. Therefore, both fossil fuels (Diesel) and alternative fuels (ROMEP) were used in this study. An EGR system with a valve permeability of 20% was used to limit NOx emissions. Although the information you requested about the injection parameters is not essential, since we focus on the pollution aspects using acoustic agglomeration, the information you requested is provided below.

The engines were tested at a load of 90 Nm. The selected engine load simulates the engine running at a speed of ∼100 km/h. Experimental tests were carried out by setting a fixed engine shaft speed of n = 2000 rpm. At this shaft speed, the maximum torque of the engine to be tested was reached, and the engine operated at maximum efficiency. The tests were carried out at the EGR rate (20%). The EGR rate is determined by estimating the mass of intake air and is controlled by an EGR controller. The exhaust gas recirculation (EGR) control was disconnected from the ECU, controlling the EGR rate using an EGR controller – pulse width modulator.

The start of injection (SOI) was controlled by the Engine Electronic Control Unit (ECU), and it was a single injection strategy. During the ignition or injection timing of the tests, the fuel (SOI = 2 CAD BTDC) was controlled by the electronic control unit of the engine. The injection timing (BMEP = 0.3 MPa) injection timing was adjusted by modulating the SOI control signal in the second experimental test step.

## 3 Results and discussion

### 3.1 Analysis of the results of the measurement of the particulate concentration

**Fig. 3:**
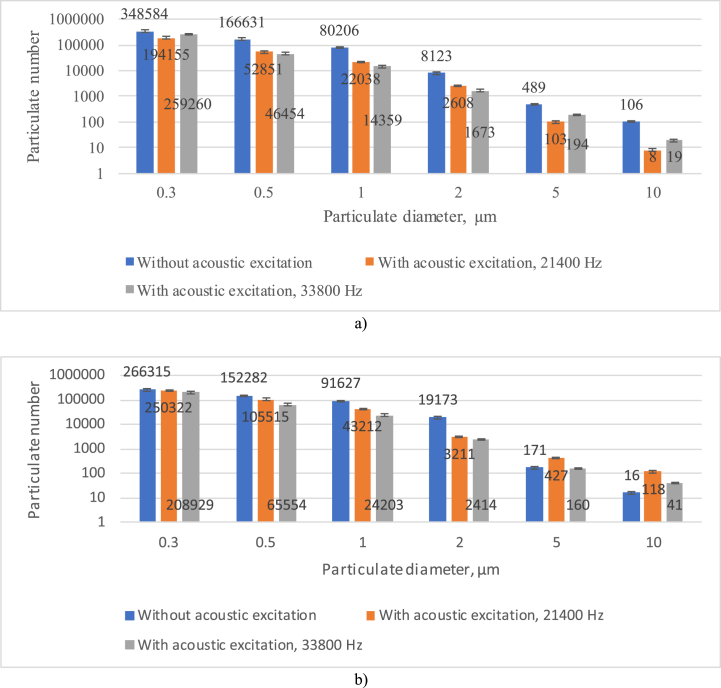
Results of particle concentration by diameter (additional conditions were EGR level 20% and Load 90 Nm): a - diesel fuel used; b - Mixture of Rapeseed Oil Methyl Esters 90% and 10% Propanol (ROMEP).

The analysis of diesel emissions (additional conditions were EGR level 20% and Load 90 Nm) and Rapeseed Oil Methyl Esters 90% and 10% Propanol (ROMEP) (additional conditions were EGR level 20% and Load 90 Nm) was the number of particulates to be determined with and without acoustic effects. The results of the particulate concentrations (averaged over six measurements for each case) are given in Fig. 3. During the measurement of the particulate concentration, the number of particles was determined with a volume of 1.42 l of aspirated air.

Assessing in Fig. 3 results of the measurements, it shows that the concentrations of particulates in the exhaust gases of diesel and ROMEP show a lower particulate matter content of 23.6, 8.6, 65.0 and 84.9% for particulate diameters of 0.3, 0.5, 5.0 and 10.0 ROMEP and 1.0 and 2.0% for particulate diameters, respectively. An increase in particles of 14.2% and 136.0%, respectively, is observed. Comparing the concentrations of particles in diesel fuel exhaust gas with and without acoustic effects, it was found that the acoustic field reduces the number of particles at all diameters considered and varies from 44.3 (particle diameter 0.3 μm) to 92.45 (particle diameter 10.0 μm) at an excitation frequency of 21400 Hz, the number of particles at all diameters considered, respectively, varies from 25.6 (particle diameter 0.3 μm) to 82.08 and 82.10 (particle diameter 10.0 and 1.0 μm). Comparing the concentrations of particles in the ROMEP exhaust gas with and without acoustic effects, it was found that the acoustic field reduces the number of small diameter particles (0.3, 0.5, 1.0 and 2.0 μm) and varies from 6.0 (particle diameter 0.3 μm) to 83.25 (particle diameter 2.0 μm) at an excitation frequency of 21400 Hz also reduces the number of small diameter particles (0.3, 0.5, 1.0, 2.0 and 5.0) to and from 6.4 (particle diameter 5.0 μm) to 87.40 (particle diameter 2.0 μm).

When assessing the patterns of PM acoustic exposure, it was found that the analysis of diesel emissions resulted in particle reductions at all PM diameters considered. The results obtained are close to those reported in the scientific literature [5,14,40]. The analysis of ROMEP fuel emissions resulted in particle reductions at PM diameters up to 2.0 μm and particle increases at PM diameters 5.0 and 10 μm. It can be concluded that the smaller particle diameters agglomerated into larger particles. When assessing the differences in PM between diesel and ROMEP fuels, it should be noted that the oxygen content is the main parameter that differs between diesel and ROMEP (0 and 10.5%, respectively). Higher oxygen content improves the combustion process and thus reduces particulate matter.

### 3.2 Exhaust gas analysis

The exhaust results (average of six measurements taken for each case) are given in Tables 2 and 3.

**Table 2:** Exhaust emissions for D100 fuel use and gas flow temperature: 28.5 °C results.

Agglomeration Conditions	Exhaust gases					
	CO, ppm	NO, ppm	NO_2_, ppm	NOx, ppm	SO_2_, ppm	O_2_, %
Without	497	380	6.5	386	0	10.65

**Table 3:** Exhaust emissions for ROMEP fuel use and gas flow temperature: 38.3 °C results.

Agglomeration Conditions	Exhaust gases					
	CO, ppm	NO, ppm	NO_2_, ppm	NOx, ppm	SO_2_, ppm	O_2_, %
With	400	310	3.5	311	1	11.31
Without	403	301	3.0	300	0	11.05

Different fuels change the combustion mode, and D100 uses slightly more oxygen for combustion. The calorific value of the fuel allows for a higher engine power output and thus increases the exhaust gas temperature, e.g., the difference between D100 and ROMEP was about 11 °C. Taking into account these energy differences, it is assumed that the emissions have to be considered independently of the influence of the acoustic agglomeration, but as a direct consequence of the type of combustion engine and the type of fuel it is powered with.

**Fig. 4:**
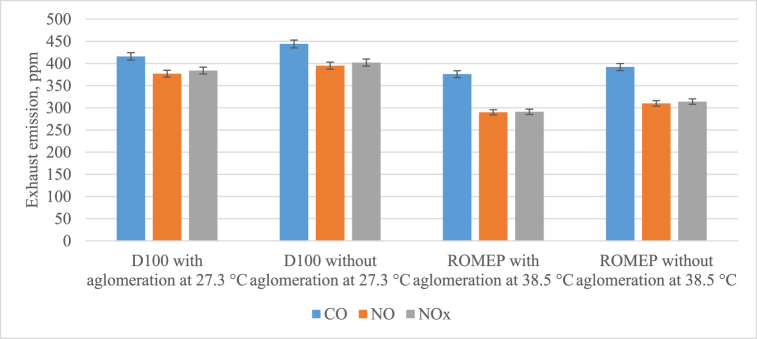
Exhaust emissions for the use of D100 (a) and ROMEP (b) fuel use at gas flow temperatures of 27.3 °C (a) and 38.5 °C (a) results.

The exhaust emissions for the use of D100 and ROMEP fuels and the gas flow temperature of 27.3 °C and 38.5 °C results are given in Fig. 4.

For secondary pollutants other than particulates, the results are presented taking into account differences in the engine exhaust components during combustion and the temperature during combustion, which changes the overall composition of the gas. Therefore, the D100 and ROMEP cases cannot be directly compared without taking these changes into account.

When pure diesel was used as fuel, the exhaust gas flow temperature reached 27.3 °C at 20% EGR and 90 Nm under load, which is more than 40% cooler than in the ROMEP case. The results do not differ significantly between 3.5% and 6.7% between the cases without and without agglomeration, so the differences can be ignored. Although the oxygen content of the gas was approximately, at about 11.2%, even at an EGR of 20%, the CO concentration found was slightly higher than in other cases, reaching 444 ppm or 998 mg/m^3^. Nitrogen oxide concentrations increased 1.3-fold, and NO_2_ share doubled. This speaks to the formation of higher-density exhaust gases, which in this study were observed only in the case of burning diesel fuel. However, in this case, no trace of sulfur dioxide was recorded, which indicates the use of sulfur-free fuel. A level of SO_2_ for all cases was very low, approximately 0–1 ppm. The oxygen content from the combustion of D100 was 0.1–0.3% higher than in the ROMEP case and the maximum value was 11.51%. The nitrogen dioxide concentration for D100 was twice that for ROMEP, due to the saturation of diesel fuel with nitrogen compounds initially in the feedstock, as opposed to the more natural content of the rapeseed oil. The maximum value of 7 ppm was achieved with agglomeration, without agglomeration - the values decreased slightly, by 0.3 ppm. When ROMEP was burned, the difference in values was not greater than 0.3 ppm and was equal to 3.4 ppm, so the difference can be neglected.

A slight increase in the combustion temperature to 28.5 °C at an analogous EGR of 20% and a load of 90 Nm (Table 3) resulted in only an increase in the CO concentration of slightly less than 500 ppm. This was also due to a 1.06-fold decrease in oxygen content of 10.65%. The concentrations of nitrogen oxides remained similar and no sulfur dioxide was detected in the flue gas stream.

### 3.3 Exhaust emissions for ROMEP fuel use and gas flow temperature 38.5 °C results are given in the analysis

When using the ROMEP fuel mixture, the exhaust gas flow temperature reached 38.5 °C at an EGR of 30% and a load of 90 Nm. The results do not differ significantly between 1.5% and 8% between cases without an agglomeration chamber and with an agglomeration chamber, so the differences can be ignored. Assuming an oxygen concentration rate of 3% for liquid combustion, there was an excess of oxygen in this case. Due to the high level of EGR, the concentration of CO as a product of incomplete combustion was up to 400 ppm, which corresponds to 890 mg/m^3^ in terms of conversion. The concentration of nitrogen oxides consisted mainly of a nitric oxide compound due to incomplete oxidation, which would increase the NO_2_ content. Traces of sulfur dioxide have been recorded because of the small amount of sulfur in the fuel mixture.

The combustion process in case the exhaust gas flow temperature reached 38.3 °C (Fig. 4) was similar to case the exhaust gas flow temperature reached 38.5 °C with a slightly lower oxygen supply.

This change increased the CO formation of the incomplete combustion product by 5% to 403 ppm. The uptake of nitrogen oxides was similar and, in this case, was slightly higher without the use of an agglomeration chamber.

## 4 Conclusions

The following effects were observed for different fuels with particulate agglomeration:

1. ROMEP-containing fuels reduce the amount of particulate matter throughout the particle range compared to fossil diesel. The additional use of agglomeration shows a decrease in particle concentration, especially in the 5 and 10 μm range.
2. The acoustic agglomeration frequency of 21400 Hz was most efficient for the extraction of ultra-fine dispersion particles using both D100 and ROMEP fuels.
3. Analysis of the results of the exhaust gas studies showed that the use of D100 fuel and agglomeration showed a slightly higher concentration of CO than in other cases, as well as an increase in nitrogen oxide emissions. The results were negligible with the use of ROMEP fuel.

An investigation would be conducted considering the most efficient case of 34 kHz or 20 kHz in more detail and to determine the intensity of the most efficient agglomeration of ultrafine particles as a function of the wave frequency in the range of 21.4–33.8 kHz. Due to the design constraints of maintaining the resonant frequency of the structure, it was not possible to maintain the other level of acoustic exposure in a physical manner that is only possible in the presence of resonance phenomena. Also, it would be appropriate to carry out future studies at higher wavelengths, e.g., above 40 kHz. An analysis of impact from the ultrasonic waves is needed explored in order to highlight larger patterns of acoustic agglomeration at different exposure frequencies, e.g., in steps of 5 kHz, for wavelengths up to 50 kHz.

Another topic of study relates to the effects of pressure changes as a result of acoustic vibrations on chemical compounds, especially pollutants, which can cause chemical reactions, the breakdown and combining of atoms or molecules.

## Author contribution statement

Kristina Kilikevičienė: Aleksandras Chlebnikovas: Jonas Matijošius: Artūras Kilikevičius: Conceived and designed the experiments; Performed the experiments; Analyzed and interpreted the data; Contributed reagents, materials, analysis tools or data; Wrote the paper.

## Data availability statement

Data will be made available on request.

## Funding

This research has received funding from the European Social Fund (project No. 09.3.3-LMT-K-712-19-0026) under a grant agreement with the Lithuanian Research Council (LMTLT).

## Declaration of competing interest

The authors declare that they have no known competing financial interests or personal relationships that could have appeared to influence the work reported in this paper.
